# Application of Electroencephalography-Based Machine Learning in Emotion Recognition: A Review

**DOI:** 10.3389/fnsys.2021.729707

**Published:** 2021-11-23

**Authors:** Jing Cai, Ruolan Xiao, Wenjie Cui, Shang Zhang, Guangda Liu

**Affiliations:** College of Instrumentation and Electrical Engineering, Jilin University, Changchun, China

**Keywords:** EEG, machine learning, emotion recognition, feature extraction, classification

## Abstract

Emotion recognition has become increasingly prominent in the medical field and human-computer interaction. When people’s emotions change under external stimuli, various physiological signals of the human body will fluctuate. Electroencephalography (EEG) is closely related to brain activity, making it possible to judge the subject’s emotional changes through EEG signals. Meanwhile, machine learning algorithms, which are good at digging out data features from a statistical perspective and making judgments, have developed by leaps and bounds. Therefore, using machine learning to extract feature vectors related to emotional states from EEG signals and constructing a classifier to separate emotions into discrete states to realize emotion recognition has a broad development prospect. This paper introduces the acquisition, preprocessing, feature extraction, and classification of EEG signals in sequence following the progress of EEG-based machine learning algorithms for emotion recognition. And it may help beginners who will use EEG-based machine learning algorithms for emotion recognition to understand the development status of this field. The journals we selected are all retrieved from the Web of Science retrieval platform. And the publication dates of most of the selected articles are concentrated in 2016–2021.

## Introduction

Emotions are the changes in people’s psychological and physiological states when they face external stimuli such as sounds, images, smells, temperature, and so on. And it plays a vital role in mental and physical health, decision-making, and social communication. To realize emotion recognition, Ekman regarded emotions as six discrete and measurable states related to physiological information, namely happy, sad, anger, fear, surprise, and disgust (Ekman, 1999; Gilda et al., 2018). Subsequent studies on emotion recognition mostly followed this emotion classification basis, but some researchers had added new emotional states, including neutral, arousal, relaxed (Bong et al., 2012; Selvaraj et al., 2013; Walter et al., 2014; Goshvarpour et al., 2017; Minhad et al., 2017; Wei et al., 2018). Some people had also provided a new classification standard for emotions, including relaxation, mental stress, physical load, mental stress combined with physical load (Mikuckas et al., 2014). The setting that emotions are discretized states makes the emotion recognition can be perfectly realized by classification in machine learning. The overall process of machine learning for emotion recognition is as follows: the subjects’ facial expressions, speech sounds, body movements (Kessous et al., 2010), electromyography (EMG), respiration (RSP) (Wei, 2013), galvanic skin response (GSR) (Tarnowski et al., 2018), blood volume pulsation (BVP), skin temperature (SKT) (Gouizi et al., 2011), photoplethysmographic (PPG) (Lee et al., 2019), electrocardiogram (ECG) (Hsu et al., 2020), heart rate (HR) (Wen et al., 2014) and electroencephalography (EEG) will appear corresponding changes when stimulated by external audio, visual, audio-visual and other stimuli. In addition to the above external factors that will affect the changes in emotions, autonomic nervous system (ANS) activity is viewed as a major component of the emotion response (Kreibig, 2010). Ekman (1992) analyzed six basic emotions by recording six ANS parameters. And Levenson (2014) discussed emotions activate different patterns of ANS response for different emotions.

The above-mentioned physiological information can be collected via specific devices, then features related to emotion states can be extracted after preprocessing the collected data, and finally, emotion recognition will be realized by classifying these features. Compared with external body changes such as facial expressions and speech sounds, the internal physiological information such as EMG, SKT, ANS, and EEG can more genuinely reflect the emotional changes of the subject due to its inability to conceal deliberately. And among the many physiological signals, there are a vast number of researches on collecting EEG, which contains relatively affluent information to recognize emotions through machine learning algorithms. Aim to classify physically disabled people and Autism children’s emotional expressions, Hassouneh et al. (2020) achieved a maximum emotion recognition rate of 87.25% using the long short-term memory (LSTM) as the classifier to EEG signals. Aim to classify Parkinson’s disease (PD) from healthy controls, Yuvaraj et al. (2014) presented a computational framework using emotional information from the brain’s electrical activity. Face the situation that the diagnosis of depression almost exclusively depends on doctor-patient communication and scale analysis, which has obvious disadvantages such as patient denial, poor sensitivity, subjective biases, and inaccuracy. Li et al. (2019) committed to automatically and accurately depression recognition using the transformation of EEG features and machine learning methods.

This paper summarizes the development of EEG-based machine learning methods for emotion recognition from four aspects: acquisition, preprocessing, feature extraction, and feature classification. It is helpful for beginners who rely upon EEG-based machine learning algorithms for emotion recognition to understand the current development of the field and then find their breakthrough points in this field.

## Acquisition of Electroencephalography Signals for Emotion Recognition

There are generally two ways to acquire EEG signals related to emotions. One way is to stimulate the subject to produce emotional changes by playing audio, video, or other materials and obtain the EEG signal through the EEG device worn by the subject. Yuvaraj et al. (2014) obtained EEG data using the Emotive EPOC 14-channel EEG wireless recording headset (Emotive Systems, Inc., San Francisco, CA) with 128 Hz sampling frequency per channel from 20 PD patients and 20 healthy by inducing the six basic emotions of happiness, sadness, fear, anger, surprise, and disgust using multimodal (audio and visual) stimuli. Bhatti et al. (2016) used music tracks as stimuli to evoke different emotions and created a new dataset of EEG signals in response to audio music tracks using the single-channel EEG headset (Neurosky) with a sampling rate 512 Hz. Chai et al. (2016) recorded EEG signals related to audio-visual stimuli using a Biosemi Active Two system. And EEG signals were digitized by a 24-bit analog-digital converter with a 512 Hz sampling rate. Chen et al. (2018) used a 16-lead Emotiv brainwave instrument (14 of which were EEG acquisition channels and 2 of which were reference electrodes) at a frequency of 128 Hz. Later, Seo et al. (2019) used a video stimulus to evoke boredom and non-boredom and collected EEG data using the Muse EEG headband from 28 Korean adult participants. And Li et al. (2019) conducted an experiment based on emotional face stimuli and recorded 28 subjects’ EEG data from 128-channel HydroCel Geodesic Sensor Net by Net Station software. In Hou et al. (2020), the Cerebus system (Blackrock Microsystems, United States) was used to collect EEG data at a 1 kHz sampling rate using a 32-channel EEG cap. In the same year, Maeng et al. (2020) introduced a new multimodal dataset via Biopac’s M150 equipment called MERTI-Apps based on Asian physiological signals. And Gupta et al. (2020) used an HTC Vive VR display to enable participants to interact with immersive 360° videos in VR and collected EEG signals using a 16-channel OpenBCI EEG Cap with a 125 Hz sampling frequency. Later, Keelawat et al. (2021) acquired EEG data based on a Waveguard EEG cap with a 250 Hz sampling rate from 12 students from Osaka University, to whom song samples were presented. What’s more, to effectively collect EEG signals, the attachment position of electrodes for EEG equipment in many studies follows the international 10–20 system (Chai et al., 2016; Seo et al., 2019; Hou et al., 2020; Huang, 2021).

Another way is to use the existing, well-known database in the field of emotion recognition based on EEG, including DEAP (Izquierdo-Reyes et al., 2018), MAHNOB-HCI (Izquierdo-Reyes et al., 2018), GAMEEMO (Özerdem and Polat, 2017), SEED (Lu et al., 2020), LUMED (Cimtay and Ekmekcioglu, 2020), AMIGOS (Galvão et al., 2021), and DREAMER (Galvão et al., 2021). After obtaining the original EEG signal related to emotion states, the following operation is to preprocess the EEG signal to improve the quality of the EEG data.

## Preprocessing Method of Electroencephalography Signal

The raw EEG data collected through EEG equipment is mixed with electronic equipment noise, as well as potential artifacts of electrooculography (EOG), electromyogram (EMG), respiration and body movements. Therefore, a series of preprocessing operations are usually performed before the feature extraction of the EEG signal to improve the signal-to-noise ratio.

Bandpass filters are used by most research institutes as a simple and effective noise removal method. However, since there is no precise regulation on the effective frequency band in the EEG signal, the bandpass filters used in different studies had different cutoff frequencies. Generally, the purpose of setting the low cutoff frequency at about 4 Hz (Özerdem and Polat, 2017; Chao et al., 2018; Pane et al., 2019; Yin et al., 2020) was to remove electrooculography (EOG) artifacts (0–4 Hz) and potential artifacts of respiration and body movements within 0–3 Hz. While some documents set the low cutoff frequency at about 1 Hz (Yuvaraj et al., 2014; Bhatti et al., 2016; Liang et al., 2019; Hou et al., 2020; Keelawat et al., 2021), the purpose of which was to remove the baseline drift (DC component) in the EEG signal and the 1/f noise introduced by the acquire equipment. On the other hand, for high cutoff frequency, most researchers set it to about 45 Hz (Kessous et al., 2010; Yuvaraj et al., 2014; Liang et al., 2019; Yin et al., 2020) to remove the other artifact noises at the high frequencies. While, some recent studies (Hou et al., 2020; Lu et al., 2020; Rahman et al., 2020) set it around 70–75 Hz to preserve more emotion-related features among the EEG to improve the accuracy of emotion recognition.

In addition to using bandpass filters for noise suppression, scholars have also adopted many other excellent methods for preprocessing EEG signals. For example, in the work of Aguiñaga and Ramirez (2018), the Laplacian filter described by Murugappan (2012) was implemented to mitigate the problem that EEG signals were naturally contaminated with noise and artifacts. And then, a blind source separation (BSS) algorithm was implemented to remove redundancy between active elements meanwhile preserve information of non-active elements. And in the study of Chen et al. (2018), the independent component analysis (ICA) was used to suppress noise. Furthermore, the study conducted in Cimtay and Ekmekcioglu (2020) compared three types of smoothing filters (smooth filter, median filter, and Savitzky-Golay) on EEG data and concluded that the most useful filter was the classical Savitzky-Golaly which smoothed the data without distorting the shape of the waves. And the main contribution of Alhalaseh and Alasasfeh (2020) relied on using empirical mode decomposition/intrinsic mode functions (EMD/IMF) and variational mode decomposition (VMD) for signal processing purposes. Besides, Keelawat et al. (2021) used EEGLAB, an open-source MATLAB environment for EEG processing, to remove contaminated artifacts based on ICA.

In addition to removing noise and artifacts, there are other tasks to be done in the preprocessing process. Since the effective frequency band of the EEG signal does not exceed 75 Hz, while the sampling rate of some acquisition devices was even as high as 1,000 Hz, far exceeding the required sampling rate, down-sampling was usually required to reduce the amount of data and increase the execution rate of the algorithm (Chao et al., 2018; Rahman et al., 2020). Besides, to correlate EEG data with brain events easily, the continuously recorded EEG data were usually segmented with time windows of different lengths according to the timestamp of occurrence (Cimtay and Ekmekcioglu, 2020). In addition, considering that the EEG signal is composed of different rhythmic components, including Delta rhythm (< 3 Hz), Theta rhythm (4–7 Hz), Alpha rhythm (8–12 Hz), Beta rhythm (13–30 Hz), and Gamma rhythm (>31 Hz), some studies used bandpass filters to separate the rhythm components in the preprocessing stage to facilitate later feature extraction (Yulita et al., 2019).

## Feature Extraction of Emotion-Related Electroencephalography Signals

Feature extraction is the algorithm of extracting the specific characteristic features from the EEG signals. These distinctive features describe each emotion in a unique way. The complexity of the emotion recognition is also reduced when the complex input signal is converted into a crisp dataset (Hemanth et al., 2018). Ten features from the time domain, frequency domain, and wavelet domain are usually extracted. Features in the frequency domain are including power spectral density (PSD). Features belonging to the time domain include latency to amplitude ratio (LAR), peak-to-peak value, kurtosis, mean value, peak-to-peak time window, and signal power. And features from the wavelet domain are including entropy and energy (Bhatti et al., 2016). Besides, fractal dimension and statistical features were used by Nawaz et al. (2020). And several non-linear features such as correlation dimension (CD), approximate entropy (AP), largest Lyapunov exponent (LLE), higher-order spectra (HOS), and Hurst exponent (HE) had been used widely to characterize the emotional EEG signal (Balli and Palaniappan, 2010; Chua et al., 2011).

To extract features related to emotional states from EEG signals, a large number of researches on feature extraction algorithms have emerged. Chai et al. (2016) proposed a novel feature extraction method called the subspace alignment auto-encoder (SAAE), which combined an auto-encoder network and a subspace alignment solution in a unified framework and took advantage of both non-linear transformation and a consistency constraint. And Özerdem and Polat (2017) used Discrete wavelet transform (DWT) for feature extraction from EEG signals. Later, Li et al. (2018) organized differential entropy features from different channels as two-dimensional maps to train the hierarchical convolutional neural network (HCNN). In the same year, Izquierdo-Reyes et al. (2018) applied the Welch algorithm to estimate the PSD of each EEG channel, using a Hanning window of 128 samples. Soroush et al. (2018) extracted non-linear features from EEG data, and they suggested feature variability through time intervals instead of absolute values of features. What’s more, discriminant features were selected using the genetic algorithm (GA). And Chen et al. (2018) leveraged EMD to obtain several intrinsic eigenmode functions and Approximation Entropy (AE) of the first four IMFs as features from EEG signals for learning and recognition. Later, In Chao et al. (2019), the frequency domain, frequency band characteristics, and spatial characteristics of the multichannel EEG signals were combined to construct the multiband feature matrix (MFM). Consider that the rhythmic patterns of an EEG series could differ between subjects and between different mental states of the same subject, Liang et al. (2019) used a segment-based feature extraction method to obtain EEG features in three domains (frequency, time, and wavelet). In Li et al. (2019), the PSD and activity were extracted as original features using the Auto-regress model and Hjorth algorithm with different time windows. And Qing et al. (2019) used the autoencoder to further process the differential feature to improve the discriminative power of the features. Besides, Yulita et al. (2019) used principal component analysis (PCA) to change most of the original variables that correlate with each other into a set of variables that are smaller and mutually independent. Later Alhalaseh and Alasasfeh (2020) used entropy and Higuchi’s fractal dimension (HFD) in the feature extraction stage. And Salankar et al. (2021) first adapted EMD to decomposes the signals into several oscillatory IMF and then extracted features including area, mean, and central tendency measure of the elliptical region from second-order difference plots (SODP). In the same year, Wang et al. (2021) proposed an emotion quantification analysis (EQA) method, which was conducted based on the emotional similarity quantification (ESQ) algorithm in which each emotion was mapped in the valence-arousal domains according to the emotional similarity matrixes.

After feature extraction, some studies also reduced the feature space by feature selection (FS) technique to avoid over-specification using large number of extracted features and to make the feature extraction feasible online. In study of Jirayucharoensak et al. (2014), the input features of the deep learning network (DLN) were power spectral densities of 32-channel EEG signals from 32 subjects. To alleviate the overfitting problem, PCA was applied to extract the most important components of initial input features. Later, Rahman et al. (2020) implemented spatial PCA to reduce signal dimensionality and to select suitable features based on the t-statistical inferences. And Zhang et al. (2020) proposed a shared-subspace feature elimination (SSFE) approach to identify EEG variables with common characteristics across multiple individuals. Yin et al. (2020) proposed a new locally robust feature selection (LRFS) method to determine generalizable features of EEG within several subsets of accessible subjects. Besides, Maeng et al. (2020) used GA to determine the active feature group from the extracted features. Also, other FS algorithms, including correlation ratio (CR), mutual information (MI), and random forest (RF), were used in Suzuki et al. (2021). After extracting the emotional state-related feature vectors from the EEG signal, the next important step is to classify these features to achieve emotion recognition.

## Classification of Emotion-Related Electroencephalography Signals

The concept of classification is to construct a classifier based on existing data. The classifier is a general term for the methods of classifying samples, and for emotion recognition using EEG signals, it is a crucial part, which takes the features extracted in the above process as input to complete the recognition of the emotional states.

Many classifiers have been implemented to help emotion recognition, including Support Vector Machine (SVM), multilayer perceptron (MLP), Circular Back Propagation Neural Network (CBPN), Deep Kohonen Neural Network (DKNN), deep belief networks with glia chains (DBN-GCs), artificial neural network (ANN), linear discriminant analysis (LDA), capsule network (CapsNet), convolutional neural network (CNN), multi-scale frequency bands ensemble learning (MSFBEL) and so on. And their emotion recognition accuracies are listed in Table 1.

**TABLE 1:** Classifiers and their performance.

**Classification Item**	**Author**	**Model**	**Accuracy (%)**
Arousal and valence	Jirayucharoensak et al., 2014	DLN	Arousal: 46.03 Valence: 49.52
	Choi and Kim, 2018	LSTM	Arousal: 74.65 Valence: 78.00
	Chao et al., 2018	DBN-GCs	Arousal: 75.92 Valence: 76.83
	Maeng et al., 2020	GA-LSTM	Arousal: 94.8 Valence: 91.3
	Keelawat et al., 2021	CNN	Arousal: 56.85 Valence: 73.34
Arousal, valence, and dominance	Chao et al., 2019	CapsNet	Arousal: 68.285 Valence: 66.73 Dominance: 67.25
Positive and negative	Özerdem and Polat, 2017	MLP	77.14
	Lu et al., 2020	SVM	85.11
Positive, negative, and neutral	Li et al., 2018	HCNN	97
	Rahman et al., 2020	ANN	86.57 ± 4.08
	Wang et al., 2020	CNN	90.59
Boredom and non-boredom	Seo et al., 2019	KNN	86.73
Pleasand and unpleasand	Gupta et al., 2020	KNN, SVM	KNN:96.5 SVM:83.7
Happy, calm, sad, and fear	Chen et al., 2018	DBN-SVM	87.32
Happy, sad, angry, and astounded	Li et al., 2019	SVM	89.02
Happy, angry, sad, and relaxed	Pane et al., 2019	RF	75.6
	Kessous et al., 2010	DKNN, CBPN	95–98
Sad, disgust, angry, and surprise	Sakalle et al., 2021	LSTM	94.12
Happy, fear, sad, and neutral	Galvão et al., 2021	MSFBEL	74.22
Happy, sad, surprise, fear, disgust, and angry	Hassouneh et al., 2020	LSTM	87.25

Liu et al. (2020) by combining the CNN, SAE, and DNN and training them separately, the proposed network is shown as an efficient method with a faster convergence than the conventional CNN. And, for the SEED dataset, the best recognition accuracy reaches 96.77%. Topic and Russo (2021) propose a new model for emotion recognition based on the topographic (TOPO-FM) and holographic (HOLO-FM) representation of EEG signal characteristics. Experimental results show that the proposed methods can improve the emotion recognition rate on the different size datasets.

Unlike researches listed in Table 1, which only identified a limited set of emotional states (e.g., happiness, sadness, anger, etc.), Galvão et al. (2021) were dedicated to predicting the exact values of valence and arousal in a subject-independent scenario. The systematic analysis revealed that the best prediction model was a KNN regressor (*K* = 1) with Manhattan distance, features from the alpha, beta, gamma bands, and the differential asymmetry from the alpha band. Results, using the DEAP, AMIGOS, and DREAMER datasets, showed that this model could predict valence and arousal values with a low error (MAE < 0.06, RMSE < 0.16).

## Conclusion and Discussion

To improve the accuracy of EEG signal-based machine learning algorithms in emotion recognition, researchers have made a lot of efforts in the acquisition, preprocessing, feature extraction, and classification of EEG signals. From the above summary, it can be found that the current stage of emotion recognition based on machine learning is mainly focused on the improvement of accuracy. What’s more, some combinations of feature extraction algorithms and classifiers can even achieve 100% accuracy in the two-classification of emotion recognition. And we believe that the following two goals that need to be achieved in emotion recognition based on machine learning are: (1) Perception of smaller changes in emotion; (2). Reduction in the complexity of emotion recognition algorithms so that the algorithm can be transplanted to wearable devices to realize real-time emotion recognition.

## Author Contributions

All authors listed have made a substantial, direct and intellectual contribution to the work, and approved it for publication.

## Conflict of Interest

The authors declare that the research was conducted in the absence of any commercial or financial relationships that could be construed as a potential conflict of interest.

## Publisher’s Note

All claims expressed in this article are solely those of the authors and do not necessarily represent those of their affiliated organizations, or those of the publisher, the editors and the reviewers. Any product that may be evaluated in this article, or claim that may be made by its manufacturer, is not guaranteed or endorsed by the publisher.
